# Multilayered Epistemic Disruption in AI-Driven Health Misinformation: Conceptual Framework and Viewpoint

**DOI:** 10.2196/96664

**Published:** 2026-08-20

**Authors:** Muzaffer Malkoç

**Affiliations:** 1 Istanbul Medipol University, Kavacık Güney Kampüsü, Göztepe Mah., Beykoz, Istanbul, 34810, Türkiye, 90 5334162689

**Keywords:** health misinformation, generative artificial intelligence, large language models, deepfakes, infodemiology, epistemic trust, health communication, conceptual framework, public health policy

## Abstract

Generative AI has transformed the health information ecosystem by enabling scalable, sophisticated health misinformation production at near-zero marginal cost. Current literature addresses AI’s role in health misinformation predominantly through a binary threat detection framework, systematically overlooking the structural, multilayered mechanisms through which AI simultaneously embeds false claims across intersecting human trust systems. This paper introduces the Multilayered Epistemic Disruption Framework (MEDF), which conceptualizes how AI-driven health misinformation structurally undermines public trust through four interdependent layers of cognitive and institutional disruption: discursive (clinical language shielding: fluent medical terminology and fabricated citations deployed as credibility signals), biometric (embodied authority transfer: deepfake appropriation of real clinicians' faces and voices), temporal (the synthetic chorus effect: near-simultaneous fabrication of apparently independent corroborating sources), and systemic (structural epistemic erosion: cumulative macro-level collapse of trust in medical institutions). Adopting a socioecological and structural epistemic approach, this viewpoint synthesizes empirical findings from communication psychology, medical sociology, and digital infodemiology, and the MEDF is explicitly positioned relative to established health communication frameworks, including the i-frame and s-frame distinction (individual-level vs system-level intervention targets) and socioecological infodemic models, with each construct's novelty defined in relation to adjacent concepts in prior literature. The MEDF proposes that AI-driven health misinformation is distinctively dangerous due to its capacity to exploit variable individual receptivity to medical authority claims and to simultaneously lower epistemic thresholds across multiple trust layers. Population-level data indicate that individuals who frequently encounter health misinformation on social media are 1.66 times more likely to report systemic distrust of health care institutions (odds ratio 1.66, 95% CI 1.11-2.48). Perceptual studies document that listeners correctly identify AI-generated voice clones only about 60% of the time and perceive a cloned voice as identical to its real counterpart in approximately 80% of trials. Existing defenses—including Content Provenance and Authenticity standards, automated deepfake detection (showing area under the curve drops of up to 50% under real-world conditions), and prebunking interventions—are shown to address only subsets of the proposed cascade, leaving temporal and systemic layers substantially unmitigated. Four testable hypotheses are advanced for empirical validation. Addressing AI-driven health misinformation requires moving beyond individual-level i-frame interventions toward structural, s-frame policy responses calibrated to each layer of the MEDF cascade. Policymakers and platforms must implement source identity verification, clinician biometric protection protocols, cross-platform ecosystem governance, and proactive trust infrastructure, with particular urgency in lower- and middle-income country contexts where regulatory capacity and platform oversight are most limited.

## Introduction: Epistemic Transformation and the Research Gap

The production and consumption of health information has historically been grounded in a robust institutional framework. Medical authority has been constructed through education, licensure, and professional accountability mechanisms, providing an epistemic assurance function for the credibility of health knowledge. The transformation of information ecosystems by algorithmic interfaces, accelerated by the proliferation of generative AI (GenAI), has brought this framework to a qualitative breaking point. The “infodemic” concept formulated by the World Health Organization during the COVID-19 pandemic foreshadowed this rupture; yet GenAI has fundamentally altered both the production logic and the scale of infodemics.

The existing literature largely addresses AI’s role in health misinformation across two axes: threat vectors (hallucinations, deepfakes, and propaganda automation) and defensive opportunities (early warning systems, real-time verification, and health literacy). These axes are sometimes consolidated under the rubric of a “dual role,” emphasizing that AI can both generate and detect misinformation through the same technical infrastructure. A recent scoping review theorized this paradox through the concept of “epistemic ambivalence”—AI’s potential to simultaneously construct and erode public knowledge [1]. However, this framing leaves a fundamental question unresolved: why is AI-driven health misinformation qualitatively distinct from other forms of misinformation?

This viewpoint’s central argument is as follows: the danger of AI in the health information ecosystem lies not in generating false information per se, but in its capacity to embed that false information simultaneously across multiple layers of human trust systems. This capacity operates through clinical language shielding (false claims wrapped in fluent medical terminology and fabricated citations), embodied authority transfer (deepfaked clinician faces and voices carrying claims their real counterparts never made), the synthetic chorus effect (the near-instantaneous fabrication of apparently independent corroborating sources), and structural epistemic erosion (the cumulative collapse of trust in medical institutions). Each layer amplifies the preceding one, producing cumulative harm that exceeds the sum of individual layers.

This framework is specific to the health domain. In political or commercial misinformation, audiences are conditioned to approach sources with some degree of skepticism. In health information, the opposite dynamic obtains: health information seekers frequently exhibit heightened receptivity to claims presented with apparent medical authority—a term preferred over more reductive framings of uniform vulnerability, in line with current National Academies of Sciences, Engineering, and Medicine guidance [2]. This receptivity is not homogeneous: empirical research documents meaningful variation in individual susceptibility across populations and contexts. Chronic disease patients show direct associations between health-related social media use and cyberchondria—excessive anxiety and false health beliefs mediated by negative affect [3]. Heightened receptivity is further documented among older adults [4], individuals experiencing depression [5], and populations experiencing psychological distress or loneliness [6]. While institutional trust in medical authority provides a powerful baseline orientation for health information seeking, this trust is neither universal nor culturally invariant: cross-cultural research documents substantial variation in public deference to medical authority, structured primarily by societal power distance and cultural egalitarianism [7,8]. Under the structural and cultural conditions where such institutional trust does operate, AI systematically targets and exploits it.

This paper is presented as a viewpoint and framework piece. The framework is a conceptual proposal that has not yet undergone empirical validation; testable hypotheses have been developed for each layer and future research agendas identified. The aim of this paper is to introduce the Multilayered Epistemic Disruption Framework (MEDF), which conceptualizes how AI-driven health misinformation structurally undermines public trust through four interdependent layers of cognitive and institutional disruption. Moving beyond binary threat–opportunity framing, the MEDF maps how generative technologies alter the structural conditions of health communication and derives layer-specific policy interventions and empirical research agendas. The structure is as follows: the Core Constructs section provides a glossary of specialized cross-disciplinary terms (Table 1) and formal definitions of the four layers; the Theoretical Framework section addresses conceptual positioning and construct originality; four subsequent sections elaborate each layer in detail; the paper then evaluates existing defense mechanisms, presents policy recommendations, and closes with testable hypotheses, conclusions, and limitations.

**Table 1.** Working definitions of specialized cross-disciplinary terms used in this paper.

Term	Working definition
Machine heuristic	The cognitive shortcut by which users automatically attribute objectivity and accuracy to machine-generated content [9].
Truth-default bias	The default tendency to presume that communication is honest unless suspicion is actively triggered, recently extended to human responses to generative AI content [10].
Liar’s dividend	The benefit accruing to dishonest actors when public awareness of deepfakes allows authentic evidence to be dismissed as fabricated [11].
Epistemic trust	In the sense of Fonagy and colleagues: an individual’s willingness to accept new communicated information as trustworthy, generalizable, and relevant to the self [12].
i-frame/s-frame distinction	The contrast between interventions targeting individual cognition and behavior (i-frame) and interventions targeting the systemic rules and structures within which individuals act (s-frame) [13].
Need for cognitive closure	The motivated desire to reach a firm answer and avoid ambiguity, leading individuals to seize on early explanations and then freeze on them [14].
Dual coding theory	The theory that information encoded simultaneously through verbal and visual channels leaves stronger memory traces than single-channel encoding [15].
Illusory truth effect	The increase in the perceived truth of a statement produced by repeated exposure, largely independent of source credibility [16].
Epistemic ambivalence	Generative AI’s paradoxical capacity to simultaneously construct and erode public knowledge [1].
Astroturfing and coordinated inauthentic behavior	The manual orchestration of ostensibly independent accounts and messages to simulate organic grassroots consensus, typically for political or commercial ends.

## Core Constructs: Definitions and Architecture of the MEDF

This viewpoint draws on specialized concepts from clinical medicine, public health, informatics, and communication research. Table 1 provides working definitions of these cross-disciplinary terms together with their source literature; the four MEDF constructs themselves are formally defined in the remainder of this section.

The MEDF is formally defined as an analytic framework that conceptualizes AI-driven health misinformation not as a content problem—discrete false claims to be detected and corrected—but as a structural process operating simultaneously across four distinct layers of the trust systems through which people assess health information. Its unit of analysis is the information ecosystem rather than the individual message or user, and its purpose is twofold: to differentiate four mechanisms of epistemic disruption that require different points of intervention, and to articulate a testable hypothesis about how these mechanisms compound one another. The MEDF is not an empirically validated causal model, nor a detection instrument; it is a conceptual lens whose propositions are advanced for empirical evaluation. The four constructs are formally defined here, before their theoretical positioning and elaboration in subsequent sections. They are ordered analytically from micro-level linguistic manipulation to macro-level institutional erosion; this ordering reflects the proposed cascade logic, not an established temporal sequence.

1. Discursive layer—clinical language shielding: the mechanism by which inaccurate, unproven, or fabricated health claims are embedded within sophisticated medical terminology, pseudoclinical reasoning, and structurally plausible but distorted or fabricated citations. The professional linguistic frame itself functions as a deceptive credibility signal, bypassing the user’s content-level evaluation capacity.
2. Biometric layer—embodied authority transfer: the digital hijacking and weaponization of a verified medical professional’s physical identity—including voice clones, facial expressions, and somatic cues via deepfakes—to disseminate health claims the clinician never authorized or made.
3. Temporal layer—the synthetic chorus effect: the automated, near-instantaneous creation of a dense, cross-channel information infrastructure—including clone websites, fabricated patient testimonials, synthetic social media profiles, and video content—centered around a single false claim, creating a persuasive illusion of independent consensus within hours.
4. Systemic layer—structural epistemic erosion: the macro-level degradation of public trust in real medical authorities and institutions, occurring as the pervasive presence of hyper-realistic health deepfakes produces cognitive fatigue and chronic skepticism toward authentic health communication—what Chesney and Citron [11] termed the liar’s dividend.

**Figure 1.**
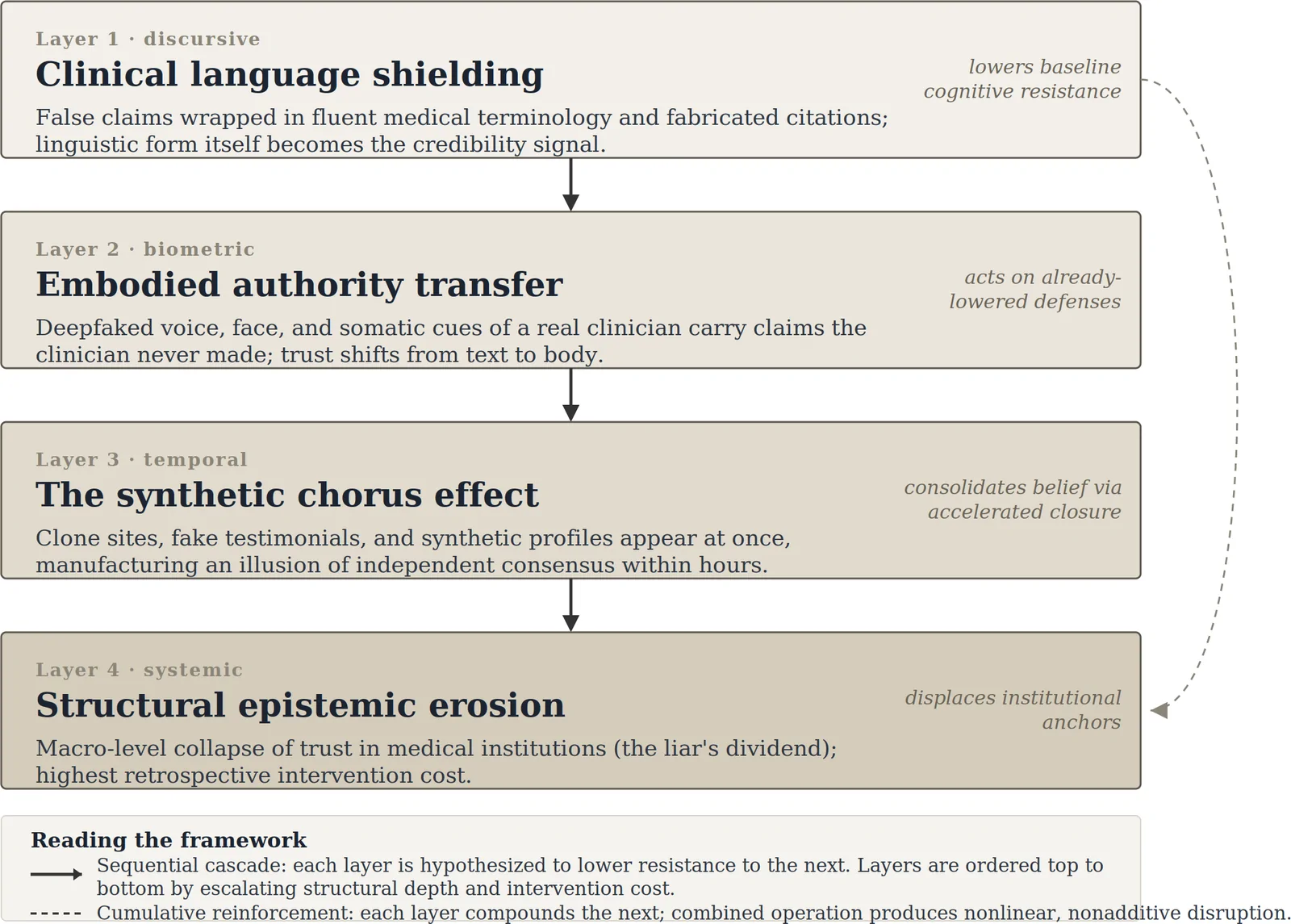
The Multilayered Epistemic Disruption Framework (MEDF): a proposed cascade across four interdependent layers of health-information trust. The cascade is a theoretical proposition advanced for empirical testing and is not yet experimentally validated.

The structural logic of the MEDF posits a sequential cascade: clinical language shielding (layer 1) is hypothesized to lower baseline cognitive resistance, rendering the user more susceptible to biometric manipulation (layer 2), which is reinforced by fabricated consensus (layer 3), ultimately contributing to systemic institutional distrust (layer 4). This cascade, if empirically confirmed, would produce harm that is nonadditive. Each layer can cause harm independently; the cascade logic constitutes the framework’s most contestable and most important empirical claim, elaborated in the Interlayer Relationships section below. Figure 1 illustrates the framework’s structure, the sequential cascade between layers, and the cumulative reinforcement dynamic. Each layer is hypothesized to lower the user’s resistance to the next (solid arrows), while their combined operation produces nonlinear, cumulative disruption (dashed reinforcement). Table 2 differentiates the four constructs by their primary trust target, core mechanism, theoretical basis, and intervention point.

**Table 2.** Differentiation of the four MEDF^a^ constructs by trust target, mechanism, theoretical basis, and intervention point.

Layer and construct	Primary trust target	Core mechanism	Principal theoretical basis	Primary intervention point
Layer 1: discursive: clinical language shielding	Linguistic markers of clinical competence	False claims embedded in fluent medical terminology and fabricated citations	Machine heuristic [9]; truth-default bias [10]	Source identity verification
Layer 2: biometric: embodied authority transfer	Embodied identity of recognized clinicians	Deepfaked face and voice carry claims the clinician never made	Deepfake perception research [17]	Clinician identity protection
Layer 3: temporal: synthetic chorus effect	Perceived independence and consensus of sources	Near-instantaneous multi-channel fabrication of corroborating sources	Source multiplicity [16,18]; need for cognitive closure [14]	Ecosystem-level governance
Layer 4: systemic: structural epistemic erosion	Institutional credibility of medical authority	Pervasive synthetic content induces chronic skepticism toward authentic communication	Liar’s dividend [11]; posttruth distrust dynamics [19]	Proactive trust infrastructure

a MEDF: Multilayered Epistemic Disruption Framework.

## Theoretical Framework and Conceptual Positioning

### Gap in the Literature and Conceptual Positioning

The MEDF is distinguished from prior work by 3 features: it treats threat and defense as two faces of one mechanism rather than separate lists; it defines the layers as independent but interconnected, such that simultaneous operation yields nonlinear rather than additive harm; and it is domain-specific. Layered frameworks exist in general disinformation research, but none systematically theorizes the vulnerability dynamics particular to health—the clinical language shield, the lowered epistemic threshold that accompanies patient anxiety, and the reflex of deference to clinical authority.

The closest conceptual neighbors in the literature are as follows:

1. Sundar’s machine heuristic [9] describes the automatic trust users extend to machine-generated content and supplies the psychological foundation for clinical language shielding. The fourth layer builds on the liar’s dividend [11]. The epistemic trust literature by Fonagy et al [12] provides grounding for understanding trust mechanisms in health contexts. Park and Nan’s [1] epistemic ambivalence offers the first systematic theorization of AI’s paradoxical capacity to both construct and erode public knowledge. These works are the MEDF’s predecessors; none of them, however, integrates these mechanisms into a unified framework specific to health misinformation.
2. A word on conceptual boundaries: clinical language shielding differs from the “generative illusion” [20] and “authority cue” concepts in that those capture general large language model (LLM) competence illusions, while clinical language shielding addresses the health-specific version of that illusion and its interaction with patient vulnerability. Likewise, the synthetic chorus effect differs from astroturfing and coordinated inauthentic behavior in that those concepts rest on manual coordination and political motivation; the synthetic chorus effect describes a low-cost, near-instantaneous multichannel production capacity now accessible to individual actors, and its specific consequences in health contexts.
3. Positioning within existing frameworks: a foundational distinction in recent behavioral public policy and health communication research is the contrast between the i-frame and the s-frame [13]. The i-frame locates solutions at the level of individual cognition and behavior; the s-frame addresses the structural, systemic conditions that shape an information ecosystem. Traditional interventions against health misinformation—individual media literacy campaigns, localized fact-checking, and content labeling—operate within the i-frame. The MEDF is explicitly an s-frame model: its central argument is that the primary harm of GenAI does not stem from individual users being deceived case by case, but from the systematic restructuring of the digital environment in ways that lower epistemic thresholds for all users simultaneously.
4. Established public health frameworks for infodemic management provide valuable structural precedents. The 4-I Fact Framework [21] offers a structured approach to information assessment and institutional trust rebuilding. Socioecological and environmental health models [22] have mapped how misinformation circulates at micro, meso, and macro levels. These frameworks were, however, largely developed for pre-GenAI disinformation dynamics or manual, human-coordinated campaigns. The MEDF builds on their structural orientation while addressing three specific dynamics outside their scope: ecosystem-level automation at near-zero marginal cost; the weaponization of clinical epistemology; and a nonlinear cascade logic in which each layer actively degrades resistance to subsequent layers.
5. From structural approaches to epistemic injustice [23], the MEDF draws the insight that epistemic harm operates not only through individual deception but through the systematic undermining of the institutional structures through which credibility is assessed and granted. Applied to AI-mediated health communication, what is eroded is not merely individual belief, but the structural conditions under which clinical authority can function as a credibility signal at all.
6. Construct originality: each of the MEDF’s four constructs requires explicit positioning relative to adjacent concepts in prior literature. The locus of the framework’s novelty claim should first be stated precisely: although the constructs vary in their distance from existing concepts, the MEDF’s primary contribution resides not in any single component but in the interaction architecture—the integration of four mechanisms into a unified, health-specific structure with a hypothesized compounding logic and a layer-specific intervention mapping. Within that architecture, the individual constructs are positioned as follows. Clinical language shielding is the framework’s most original analytical contribution. While analogous dynamics—medical framing as a persuasion strategy, professional language as a credibility heuristic—have been documented in health communication and contested illness research, prior literature addresses these as human-authored, limited-scale phenomena. Clinical language shielding formalizes the AI-specific version, characterized by LLM fluency, scalability, and the automated generation of fabricated clinical citations. Embodied authority transfer draws on established deepfake research; its specific contribution is formalizing biometric identity theft as a proposed public health mechanism, distinct from privacy violation or political disinformation contexts, and treating the empirical confirmation of this mechanism as an explicit research agenda item. The synthetic chorus effect is theoretically novel in combining source multiplicity, simultaneous cross-channel presence, and GenAI-enabled instantaneous deployment; existing astroturfing and coordinated inauthentic behavior frameworks, designed for manual, slower-moving operations, do not capture this dynamic. Structural epistemic erosion draws directly on the liar’s dividend [11]; the MEDF’s contribution is applying this concept to health communication and articulating its differential geographic and institutional impact.

### Interlayer Relationships: The Cumulative Disruption Dynamic

The question of how the four layers reinforce one another is the framework’s most critical, and most contestable dimension. A fair critique will note that layers overlap in practice: clinical language shielding operates inside deepfake videos; the synthetic chorus effect directly feeds structural erosion. This overlap is not a methodological problem; it is the framework’s central claim. The layers intersect. Their analytical value lies precisely in the fact that they require different points of intervention. Countering clinical language shielding calls for source authentication; countering embodied authority transfer calls for identity protection; countering the synthetic chorus effect requires ecosystem-level governance; and countering structural epistemic erosion requires proactive trust infrastructure. No single intervention can address all four simultaneously, and that is why treating AI-driven health misinformation as a single problem with a single fix has so far produced limited results.

The theoretical basis for the nonlinear, cumulative character of this harm rests on two bodies of evidence already present in the literature. First, the work by Fonagy et al [12] on epistemic trust in psychotherapeutic contexts established that trust mechanisms are hierarchically organized, with credibility assessments at one level modulating openness to information at the next. It must be stated transparently that this framework was developed to account for mentalizing processes in clinical relationships; applying it to AI-mediated health information ecosystems is a deliberate theoretical extension, and the cascade mechanism proposed here should be read as a theoretically motivated hypothesis rather than an established finding, pending direct experimental validation. The proposed dynamic is as follows: when clinical language shielding successfully mimics technical competence, it is hypothesized to lower the user’s baseline cognitive resistance; deepfake imagery then encounters a subject whose epistemic defenses have already been partially lowered; the synthetic chorus effect consolidates a belief formed under conditions of reduced resistance; and structural erosion sets in as those beliefs begin to displace institutional anchors. This proposed cascade, if empirically confirmed, would produce harm that is nonadditive.

Second, Kruglanski and Webster’s [14] need for cognitive closure establishes that once a satisfying explanation is reached, the tendency is to defend rather than reopen it. Critically, the speed at which closure is reached increases with the number of consistent signals encountered. Multichannel exposure, the defining feature of the third layer, does not merely add to the probability of belief; it accelerates the closure process in a nonlinear fashion, because each additional consistent signal reduces the cognitive resources the subject is willing to invest in continued scrutiny. The synthetic chorus effect exploits this dynamic directly. Taken together, these two frameworks supply the psychological mechanism that bridges individual layer effects to the cumulative harm the framework predicts: prior trust erosion lowers the threshold for closure, and closure accelerates with signal density in a way that no linear model can capture.

One clarification prevents misreading: the cascade is not advanced as a claim of necessary sequence. Any layer can be activated independently: a user may encounter a clinician deepfake without prior exposure to LLM-generated text, and structural erosion can be fed by the mere public awareness that synthetic content exists [11]. The framework’s claim is that when layers cooccur, their joint operation is hypothesized to produce more-than-additive harm; it is the compounding, not the ordering, that constitutes the central empirical proposition.

## Layer 1—Discursive: Clinical Language Shielding

### Mechanism and Positioning in the Literature

LLMs can command virtually any domain of expertise at high fluency, including medical terminology. That fluency, combined with the models’ inability to verify the accuracy of what they produce, generates a health-specific threat: clinical language shielding. The mechanism works as follows: a false health claim is wrapped in medical terminology, plausible-sounding clinical reasoning, and fabricated or distorted references. When a user reads the result, it is not the content they evaluate, it is the language itself that functions as the credibility signal. This explains both Sundar’s machine heuristic [9]—the automatic trust attribution users extend to machine-generated output—and the paradox identified by Markowitz and Hancock [10]: LLMs are more truth-biased than humans, which is precisely what makes their production of false claims so dangerous.

### Empirical Evidence

A 2026 Lancet Digital Health study by Omar and colleagues provided systematic documentation of this mechanism, analyzing 3.4 million prompts across 20 LLMs [24]. Testing with real hospital discharge notes, social media health myths, and 300 clinician-approved scenarios, the researchers found that existing safety filters could not reliably distinguish true from fabricated claims when those claims were wrapped in familiar clinical language. A related randomized controlled experiment involving medical students [25] found that misleading AI explanations significantly degraded diagnostic accuracy, while accurate AI explanations produced no statistically significant improvement. This asymmetry, the harm of false information outweighing the benefit of true information, is precisely what makes clinical language shielding so dangerous in health contexts.

The scope of this layer extends well beyond supplement marketing. Research on vaccine hesitancy shows that LLMs can present inaccurate claims about vaccine safety within a scientific-looking frame when safety guardrails are bypassed [26]. In oncology contexts, herbal treatments and off-protocol regimens can be legitimized through clinical terminology. The same system-instruction vulnerabilities documented by Modi et al [26] make it technically feasible to frame unproven treatments in scientifically credible language once guardrails are circumvented.

### Testable Hypothesis

Hypothesis 1 predicts that identical false claims framed in clinical terminology produce higher belief and compliance than in everyday language.

## Layer 2—Biometric: Embodied Authority Transfer

### Mechanism

Where clinical language shielding operates at the discursive level, deepfake technology carries the threat into a biometric one: it is no longer language that provides reassurance, but the body itself. A recognized physician’s face, voice, posture, and eye contact activate social recognition and parasocial trust mechanisms that operate below conscious scrutiny. This framework terms this dynamic embodied authority transfer: the weaponization of a real medical authority’s biometric identity to carry claims that authority never made. Barrington et al [17] found that people cannot reliably distinguish AI-generated voice clones from real speech: participants perceived a cloned voice to be the same as its real counterpart approximately 80% of the time, and correctly identified a voice as AI-generated only about 60% of the time. Liu et al [27] demonstrated experimentally that AI doctor avatars appearing older elicit higher trust ratings, suggesting that deepfake designers are deliberately modeling preexisting cognitive biases in their audiences.

### Documented Patterns and Topic Distribution

A Bitdefender Labs investigation covering March–May 2024 on Meta platforms identified over 1000 deepfake health videos promoting more than 40 supplement products, targeting diabetes, joint pain, vision problems, and cardiac conditions [28]. A Full Fact investigation documented networks of accounts using deepfaked academics and clinicians across TikTok (ByteDance) and Instagram (Meta Platforms, Inc) [29], and reporting in the medical press has described the same impersonation pattern across other major platforms [30]. A 2026 statement by the American Medical Association chief executive officer in STAT News [30] documented the same pattern concentrated around glucagon-like peptide-1 alternatives, weight loss pills, and diabetes treatments.

The topics targeted are not random. Diabetes, obesity, and weight management, menopause, joint pain, and antiaging products cluster around conditions characterized by chronicity, stigma, waiting-list pressure, and a perceived inadequacy of conventional care. This mirrors broader health misinformation patterns: vaccine misinformation research shows an identical vulnerability mapping, with high-anxiety and low-institutional-trust contexts as primary targets [31]. The Wellness Nest case illustrates the dynamic concretely: Professor David Taylor-Robinson’s expertise in child health was weaponized to promote menopause supplements — a deliberate mismatch between domain authority and topic that reveals how the mechanism works. Affected clinicians have had to spend hours trying to have deepfake content removed, defending both their professional reputation and their patients’ safety.

### Testable Hypothesis

Hypothesis 2 predicts belief and compliance increase from text to anonymous avatar to recognized-clinician deepfake, with the largest gap at the deepfake step.

## Layer 3—Temporal: The Synthetic Chorus Effect

### Mechanism

The synthetic chorus effect names a specific kind of skepticism erosion: the dissolution of doubt that occurs when AI constructs a comprehensive credibility infrastructure around a single claim—websites, academic-looking articles, patient testimonials, clinician blogs, social media profiles, and videos—simultaneously and within hours. This mechanism is best understood as the intersection of three well-established psychological processes, now operating in an environment their original theorists could not have anticipated. First, source multiplicity research consistently shows that information attributed to multiple independent sources is perceived as substantially more credible than the same information from a single source [16,18]. Second, repeated exposure, independent of source, increases the perceived truth of a statement: the illusory truth effect [16]. The synthetic chorus effect is analytically distinct from, and complementary to this mechanism: the illusory truth effect operates through temporal repetition of the same claim across successive encounters, whereas the chorus operates through the structural density of apparently independent corroborating sources encountered at a single moment. Repetition builds familiarity over time; the chorus manufactures consensus all at once. Third, Paivio’s [15] dual coding theory and Mayer’s [32] cognitive theory of multimedia learning demonstrate that information encoded simultaneously through verbal and audiovisual channels leaves a stronger, more durable memory trace than single-channel exposure, reinforcing both retention and subjective certainty. Add to this Kruglanski and Webster’s [14] need for cognitive closure: once a person reaches a satisfying explanation, they tend to defend that judgment rather than reopen it. Multichannel exposure accelerates that closure. The synthetic chorus effect exploits all of this at once. Every apparent source—the website, the blog, the patient story, and the video—was produced by a single actor within hours. The user perceives independent corroboration; what they are experiencing is a coordinated illusion of consensus.

### The Temporal Factor and Collapse of Defensive Capacity

The most critical feature of this layer is its speed. Traditional disinformation operations took time to build credibility: the age of a website, the history of a social media account, accumulated engagement patterns—these were all verification signals. AI eliminates that cost. A coherent, mutually reinforcing information ecosystem spanning multiple channels can now be built in hours. Even where health information consumers approach isolated videos or single promotional pages with skepticism, the framework proposes that encountering a whole system—website, blog, social media profiles, patient testimonials, and user reviews—can erode that skepticism considerably; this proposition is formalized in hypothesis 3 below.

The synthetic chorus effect’s emergence is best understood against the backdrop of how social media had already transformed information environments before GenAI. Research on online news spread established that false stories diffuse faster, more broadly, and more deeply than true ones across social networks, driven primarily by human sharing behavior [33]. Earlier analyses documented how the structural incentive architecture of social media, algorithmic amplification of engagement-maximizing content, had already converted passive information consumers into active amplifiers, a dynamic Phillips [34] described as the oxygen of amplification in the context of political disinformation. GenAI did not create this vulnerability; it automated its exploitation at a previously inaccessible scale. The concepts of astroturfing and coordinated inauthentic behavior describe the political predecessors of the synthetic chorus effect, but were developed around the manual coordination capacity of the pre-GenAI era. The DC Weekly operation’s documented increase in content production through AI use [35] illustrates the scale shift: what previously required organized human networks can now be achieved by a single actor within hours.

### Empirical Gap and Testable Hypothesis

No empirical study has directly tested the synthetic chorus effect—this is one of the paper’s most important research gap findings. Existing disinformation research predominantly addresses isolated content units. Controlled experiments measuring the effect of simultaneous multichannel presence on user trust and health behavior represent the most pressing research agenda this framework generates.

Hypothesis 3 predicts simultaneous multichannel presence raises trust nonlinearly, the gain from two to four channels exceeding that from one to two.

## Layer 4—Systemic: Structural Epistemic Erosion

### Mechanism: The Liar’s Dividend and Institutional Trust

The cumulative effect of the first three layers feeds the fourth and deepest: structural epistemic erosion. This dynamic, what Chesney and Citron [11] named the liar’s dividend, the way that the mere existence of fake content erodes trust in real content, is particularly destructive in health communication. A patient who has watched a deepfake of a doctor may approach their next real clinical consultation, or the next message from a public health authority, with a residual skepticism that was not there before. This erosion does not target individual health decisions; it undermines institutional trust in medical authority as such. A nationally representative US study of 5041 adults found that those who reported encountering substantial health misinformation on social media were 1.66 times more likely to report low trust in the health care system (odds ratio 1.66, 95% CI 1.11‐2.48) [36].

Documented cases, including patients who abandoned prescribed treatments after watching deepfake physician endorsements of alternative remedies [29,30], illustrate how medical authority can be structurally displaced. Health communication research shows that once institutional distrust is established, the effectiveness of corrective messages drops substantially [19]. On this proposed logic, the fourth layer may represent the most consequential: structural epistemic erosion carries the highest projected retrospective intervention cost.

### Epistemic Fatigue and Beyond the Global North

A second mechanism within structural erosion is epistemic fatigue: the cognitive exhaustion of constantly asking which information is real and which was generated by AI. Research shows that exposure to highly realistic disinformation weakens confidence in distinguishing truth from fabrication—a kind of cynicism some researchers have begun calling truth fatigue [37]. The authority being eroded is, as medical sociology has long documented, a historically constructed achievement rather than a natural fact: professional dominance over health knowledge and the lay–expert boundary were institutionalized over more than a century of professionalization [38,39]. Structural epistemic erosion can therefore be read as the synthetic acceleration of that construction’s reversal—an authority assembled over a century, destabilized at the pace of automated content generation. Experimental evidence is consistent with this proposed dynamic: exposure to deepfakes has been shown to generate uncertainty more readily than outright deception, and that uncertainty in turn reduces trust in news encountered on social media [40] — a cynicism pathway that structural epistemic erosion extends to the health domain.

The geographic dimension of this layer deserves attention. Countries with developed digital infrastructure but relatively weak regulatory frameworks and uneven health literacy, for example, Brazil, India, and Nigeria, represent the contexts where this framework’s predictions are likely sharpest. In lower- and middle-income countries (LMICs), deepfake detection infrastructure is limited, platform oversight is thin, and clinicians have limited capacity to monitor their own digital footprint. The MEDF’s health-domain specificity is most pronounced in these settings: where baseline institutional health distrust is already high, structural epistemic erosion can advance considerably faster.

### Testable Hypothesis

Hypothesis 4 predicts cumulative deepfake exposure reduces trust in clinicians and institutions and depresses health-seeking behavior, most sharply where baseline distrust is high.

## Evaluation of Existing Defense Mechanisms

### Content Labeling and Provenance Standards

China’s Cyberspace Administration regulations, effective September 2025, require all AI-generated content to carry labeling at both the visual and metadata level. The EU AI Act and US state-level legislation impose comparable transparency obligations. Technical standards such as C2PA (Coalition for Content Provenance and Authenticity) aim for cryptographic verification of content origin. These measures offer a partial response to layers 1 and 2, but carry two fundamental limitations.

The first is an enforcement gap: synthetic health content frequently circulates without any transparency label, as metadata can be stripped during peer-to-peer reposting and visual watermarks can be deprioritized in platform interface designs. Initiatives such as Google’s SynthID and TikTok’s C2PA integration are promising, but their effectiveness depends on platform compliance that is not yet mandatory or consistently monitored. The second limitation is that labeling does not directly target the synthetic chorus effect (Layer 3) or structural epistemic erosion (Layer 4): flagging a single content unit within a coherent ecosystem does not undo the system’s persuasive force.

### Detection Systems and Their Limitations

The World Health Organization’s EARS (early AI-supported response with social listening) platform and similar systems hold real potential for early detection of health misinformation. However, the Deepfake-Eval-2024 benchmark found that state-of-the-art deepfake detection models show area under the curve drops of up to 50% for video, 48% for audio, and 45% for images when evaluated on real-world content versus controlled datasets [41]. The synthetic chorus effect compounds the challenge further: when campaign components are distributed across platforms and appear independent of one another, algorithmic identification of their coordination is technically difficult.

### Prebunking: The Individual-Level Limitation

The demonstrated inadequacy of post hoc correction has driven interest in prebunking. Experimental evidence supports the effectiveness of gamified prebunking interventions in certain contexts [42]. Viewed through the MEDF lens, however, prebunking carries a structural limitation that the i-frame and s-frame distinction [13] makes precise: it is an i-frame intervention targeting individual cognition, whereas the synthetic chorus effect operates at the s-frame level of the information environment itself. Equipping individuals to critically evaluate isolated content units may not be enough to hold back an ecosystem-level manipulation. A person who remains skeptical of a single piece of content can still find their defenses eroded by a coherent ecosystem of dozens of apparently independent sources. Individual media literacy is necessary but cannot substitute for structural, s-frame interventions at the ecosystem level.

## Policy Recommendations

The MEDF makes a straightforward argument: because each of its four layers requires a different point of intervention, policy frameworks that address only one or two of them will leave significant damage pathways open. Consistent with the framework’s s-frame orientation [13], the recommendations below target the structural conditions of the information ecosystem rather than individual user behavior. They are derived directly from the framework’s structure and are calibrated against what existing initiatives address and what they miss.

### Source Identity Verification

An effective defense against clinical language shielding must target the source, not the language. This means developing mandatory source authentication standards for AI systems that generate health information, and expanding the availability of health communication AI models trained on peer-reviewed medical literature with strengthened safety barriers. The practical obstacles are real: platform-level compliance requires global coordination, and companies such as Meta and Google may resist such constraints as encroachments on commercial autonomy. Joint American Medical Association–World Health Organization platform standardization efforts are promising in this space but have not yet produced binding frameworks.

### Clinician Identity Protection Protocols

Recognizing clinician identity as a legally protected professional asset, as the American Medical Association has proposed, and requiring platforms to verify accounts using physician identities for health content are critical steps. The US TAKE IT DOWN Act (May 2025) mandates removal of synthetic personal content within 48 hours, but targets privacy violations rather than health misinformation. A health-specific equivalent does not yet exist. Article 50 transparency requirements in the European Union AI Act could partially close this gap, but enforcement mechanisms need strengthening. A further structural constraint follows from the framework’s own temporal logic: false content diffuses significantly faster than true content across social networks [33], and the synthetic chorus effect compresses ecosystem construction into hours, so a 48-hour removal window can intervene only after the bulk of organic dissemination has already occurred. Removal mandates are necessary, but they are temporally outpaced by the threat they target.

### Ecosystem-Level Governance

The synthetic chorus effect requires interventions that go beyond individual content moderation. Risk-scoring systems that evaluate account age, engagement patterns, and content consistency together, and shared threat intelligence mechanisms that enable detection of coordinated campaigns across platforms, are the natural policy response. Researcher–platform collaboration models such as Stanford Internet Observatory’s Spamouflage tracking offer good precedents; health-specific equivalents have not yet been institutionalized. Cross-platform threat intelligence also presupposes moderation capacity that is unevenly distributed: in the LMIC contexts where this framework predicts the sharpest effects, platform moderation resources and local-language coverage are thinnest, and operations hosted outside national jurisdictions remain largely beyond the reach of instruments such as the European Union AI Act.

### Proactive Trust Infrastructure

Structural epistemic erosion cannot be meaningfully addressed through reactive correction alone. User-friendly tools that allow clinicians to cryptographically sign authentic content (C2PA-compatible), institutional mechanisms for health organizations to mark their digital presence as verifiable, and health literacy programs redesigned to include ecosystem awareness, teaching people to recognize coordinated information systems, not just individual false claims, all belong in this layer. In LMIC contexts, building this infrastructure requires additional resources; it also offers the greatest potential impact in precisely those settings. Given the temporal limits of removal noted above, platform architectures could also explore correction mechanisms that preserve rather than delete. Because removal can feed a censorship narrative consistent with the liar’s dividend [11], and because corrective messages lose effectiveness once institutional distrust is established [19], replacing an identified synthetic impersonation with a clinician-authorized disclaimer, while leaving its distribution links intact, would convert the falsehood’s accumulated reach into a delivery vector for authentic correction. Such interception protocols remain untested and would face substantial regulatory, privacy, and platform-liability constraints; they are advanced here as a direction for design research rather than as deployable solutions.

A final consideration concerns sequencing and evaluability. These four intervention classes differ in their deployment horizons. Provenance labeling and ecosystem-level risk governance can be pursued immediately through existing instruments, C2PA standards and, in the European Union, the systemic-risk assessment obligations that the Digital Services Act already imposes on very large online platforms, whereas clinician biometric protection requires new institutional mechanisms, and proactive trust infrastructure is a long-horizon investment whose effects compound slowly. The framework’s hypotheses double as evaluation criteria: the relationship that hypothesis 4 predicts between cumulative deepfake exposure and institutional trust, for example, provides a measurable baseline against which the effectiveness of any of these interventions can be assessed over time. The recommendations are thus offered not as a finished regulatory program but as a layer-indexed agenda whose components can be prioritized by deployment horizon and monitored through the same empirical apparatus the framework proposes.

## Testable Hypotheses, Conclusions, and Limitations

### Research Agenda

Four testable hypotheses, formally stated in their respective layer sections above, are proposed to guide empirical validation of the MEDF. In summary: hypothesis 1 predicts a clinical-terminology advantage in belief and behavioral compliance for identical false claims (layer 1); hypothesis 2 predicts an ordered increase in trust from text to anonymous synthetic avatar to recognized-clinician deepfake, with the largest gap at the deepfake step (layer 2); hypothesis 3 predicts nonlinear gains in trust under simultaneous multi-channel presence (layer 3); and hypothesis 4 predicts that cumulative deepfake exposure erodes trust in real clinicians, health institutions, and health-seeking behavior, most sharply in populations with high baseline institutional distrust (layer 4).

A and B experiments are the natural methodology for testing hypothesis 1, hypothesis 2, and hypothesis 3; longitudinal survey designs for hypothesis 4; and cross-cultural comparative studies for the LMIC dimensions of hypothesis 3 and hypothesis 4. Full operational specifications for all four hypotheses are provided in Multimedia Appendix 1.

### Conclusions

This viewpoint has proposed an analytical model, the MEDF, that addresses AI’s role in health misinformation beyond the dual-role threat-opportunity framing that currently dominates the literature. The argument is that the danger does not lie in the falsehood of individual content units but in AI’s capacity to embed false information simultaneously across four distinct layers of human trust systems.

The paper makes three contributions. First, it systematically theorizes the vulnerability dynamics specific to health misinformation, clinical language shielding, embodied authority transfer, the synthetic chorus effect, and structural epistemic erosion, and explicitly differentiates each from similar concepts in adjacent literature. Second, it maps which existing defense mechanisms address which layers and which layers they leave untouched, deriving policy recommendations with an honest account of their practical constraints. Third, it acknowledges the research gap openly, advances testable hypotheses for each layer, and contributes to shaping an empirical research agenda.

### Limitations

Three limitations deserve acknowledgment. First, the MEDF is an untested theoretical framework. The synthetic chorus effect in particular, and the cumulative harm claim more broadly, are propositions awaiting controlled experimental validation. Second, the paper draws predominantly on English-language and Western-context literature. Different authority perceptions, local language dynamics, and digital literacy patterns in LMIC contexts are underrepresented—a limitation that has been flagged throughout the analysis but that comparative research will need to systematically address. Third, the documented cases come predominantly from the supplement and commercial health fraud context; systematic mapping of similar patterns in vaccine misinformation, pandemic management, and oncology would substantially extend the framework’s scope.

## Supplementary material

10.2196/96664 Multimedia Appendix 1 Full operationalization of the four MEDF hypotheses, including proposed conditions, predicted direction of effect, and indicative empirical designs for each layer.

## Abbreviations

C2PA: Coalition for Content Provenance and Authenticity
GenAI: generative AI
LLM: large language model
LMIC: lower- and middle-income countries
