# Supplementary material for: Multilayered Epistemic Disruption in AI-Driven Health Misinformation: Conceptual Framework and Viewpoint
Source: JMIR Infodemiology. 2026 Aug 20;6:e96664. doi: 10.2196/96664 (PMC13494861; doi:10.2196/96664)
Supplement: Supplementary file 1 [file infodemiology-v6-e96664-s001.docx]

# Multimedia Appendix 1. Full Operationalization of the Four MEDF Hypotheses

This appendix accompanies the Viewpoint "Multi-layered Epistemic Disruption in AI-Driven Health Misinformation: Conceptual Framework and Viewpoint." It provides the complete operational specification of the four testable hypotheses summarized in the main text. Each hypothesis corresponds to one layer of the Multi-layered Epistemic Disruption Framework (MEDF) and is stated here with its proposed conditions, predicted direction of effect, and an indicative empirical design. These specifications are advanced for empirical evaluation; the framework itself has not yet been experimentally validated.

## H1 — Discursive Layer: Clinical Language Shielding

**Hypothesis.** When an identical false health claim is presented in (a) everyday language and (b) clinical terminology, belief rates and behavioral compliance will be significantly higher for the clinical-terminology version.

**Indicative design.** A controlled between- or within-subjects experiment in which participants are exposed to semantically equivalent versions of the same false claim differing only in linguistic register (lay vs clinical), with belief and stated behavioral intention as primary outcomes.

## H2 — Biometric Layer: Embodied Authority Transfer

**Hypothesis.** When an identical false medical claim is delivered via (a) text, (b) an anonymous synthetic avatar, and (c) a deepfake of a recognized clinician, belief rates and behavioral compliance will increase in that order, with the largest gap between (b) and (c).

**Indicative design.** A three-condition experiment holding claim content constant while varying delivery modality, testing both the ordinal prediction and the specific prediction that the recognized-clinician deepfake produces a disproportionate increase relative to the anonymous avatar.

## H3 — Temporal Layer: The Synthetic Chorus Effect

**Hypothesis.** When a health claim is presented via (a) a single channel, (b) two independent channels, and (c) four simultaneous channels — website, social media, patient testimonials, and a blog — user trust and behavioral compliance will increase in a non-linear pattern, with the gap between (b) and (c) disproportionately larger than the gap between (a) and (b).

**Indicative design.** A multi-condition experiment manipulating the number of apparently independent corroborating channels, with the central test being the non-linearity of the trust response (i.e., a disproportionate increment at higher source density) rather than a simple monotonic increase.

## H4 — Systemic Layer: Structural Epistemic Erosion

**Hypothesis.** As cumulative exposure to health deepfakes increases, trust in health information from real clinicians, trust in health institutions generally, and health-seeking behavior will each decline significantly; this effect will be more pronounced in populations with higher baseline levels of institutional health distrust.

**Indicative design.** A longitudinal survey design tracking cumulative exposure against the three trust/behavior outcomes over time, with baseline institutional distrust as a moderator, complemented where feasible by cross-cultural comparison to test the differential-vulnerability prediction.

*Note. These designs are indicative rather than prescriptive; they are intended to demonstrate the falsifiability of each construct and to guide future empirical work. Cross-cultural comparative studies are particularly relevant to the lower- and middle-income country dimensions of H3 and H4.*
